# Associations between native myocardial T1 and diastolic function evaluated by PC-CMR in patients with severe aortic valve stenosis

**DOI:** 10.1186/1532-429X-17-S1-Q18

**Published:** 2015-02-03

**Authors:** Florence Pontnau, Nadjia Kachenoura, Emilie Bollache, Gilles Soulat, Golmehr Ashrafpoor, Ludivine Perdrix, Martin Graves, Valentina Zhygalina, Benoit Diebold, Jean Noel Fabiani, Elie Mousseaux

**Affiliations:** 1 Cardiology, European Hospital Georges Pompidou, Paris, France; 2 University Pierre and Marie Curie, Paris, France; 3 Radiology, European Hospital Georges Pompidou, Paris, France; 4 Cambridge University Hospital, Cambridge, UK; 5 European Hospital Georges Pompidou, Paris, France; 6 Cardiovascular Surgery, European Hospital Georges Pompidou, Paris, France

## Background

To assess the relationship between the presence of myocardial interstitial fibrosis as reflected by the increase in native T1 values and alterations in left ventricular (LV) diastolic function evaluated by phase contrast cardiac magnetic resonance (PC-CMR), in subjects with severe aortic valve stenosis (AVS).

## Methods

We studied 20 subjects (71±10 years) with severe AVS including 19 with a preserved ejection fraction. All patients underwent transthoracic echocardiogram (TTE) and cardiac magnetic resonance (CMR) exams. CMR included conventional LV systolic function and delayed enhancement evaluations as well as a native T1 mapping acquisition using the modified Look-Locker inversion recovery sequence and velocity encoding data of the transmitral inflow for the evaluation of LV diastolic function. These latter CMR data were analyzed using custom software resulting in segmental T1 values and diastolic parameters such as transmitral peak velocities (E, A), peak flow rates (Ef, Af), filling volume (FV), and myocardial peak velocities.

## Results

For all patients, TTE revealed the presence of severe AVS according to ESC criteria (aortic valve area indexed to BSA= 0.43±0.09 cm2/m2 and mean gradient 54±14mmHg). When compared to CMR data of 34 elderly controls (60±8 years) despite the preserved ejection fraction (patients=66±10%; controls=66±4%), diastolic parameters indicated an impaired LV relaxation in patients with severe AVS. Importantly, while dense fibrosis volume quantified from delayed enhancement images was not related to diastolic function parameters, a significant relation was found between native myocardial T1 values and parameters of LV filling such as: the ratio between the peak filling rate and the peak atrial rate EfMR/AfMR (r=0.51; p<0.05); the ratio between the peak atrial rate and the filling volume Af/FVMR (r=0.67; p<0.05); and the peak atrial rate Af (r=0.63; p<0.05).

## Conclusions

Interstitial myocardial fibrosis assessed non-invasively by native T1 is related to the severity of diastolic dysfunction in subjects with severe AVS.

## Funding

This study was funded by Assistance Publique des Hôpitaux de Paris.

